# Neoadjuvant Chemoimmunotherapy Combined With Node‐Sparing Radiotherapy for Clinical T3N+ Locally Advanced Esophageal Squamous Cell Carcinoma: A Prospective Single‐Arm, Phase II Study (CINSREC Trial)

**DOI:** 10.1111/1759-7714.70191

**Published:** 2025-11-18

**Authors:** Xu Zhou, Chunji Chen, Ya Zeng, Zhangru Yang, Yan Zhuo, Yukun Wang, Liye Zhang, Xuwei Cai, Xufeng Guo

**Affiliations:** ^1^ Department of Thoracic Surgery Shanghai Chest Hospital, Shanghai Jiao Tong University School of Medicine Shanghai China; ^2^ Department of Radiation Oncology Shanghai Chest Hospital, Shanghai Jiao Tong University School of Medicine Shanghai China; ^3^ School of Life Science and Technology, ShanghaiTech University, Shanghai Clinical Research and Trial Center Shanghai China

## Abstract

**Introduction:**

The promising therapeutic outcomes of neoadjuvant chemoimmunotherapy (NCIT) in locally advanced esophageal squamous cell carcinoma (ESCC) have been confirmed by multiple phase II clinical trials and are widely used in clinical practice. However, there are some cases, such as clinical T3N+ stage, that achieve poor tumor regression after receiving NCIT, reflecting the insufficient efficacy of NCIT for advanced T‐type tumors. It may be necessary to add concurrent radiotherapy to further improve the local control effect of tumor, but it also means higher adverse events and immune suppression when irradiating tumor‐draining lymph nodes. Nevertheless, node‐sparing radiotherapy can enhance the effect of NCIT with fewer adverse effects, which has been applied to other solid tumors. The aim of this study was to evaluate the safety and efficacy of NCIT combined with node‐sparing radiotherapy for clinical T3N+ locally advanced ESCC (CINSREC trial).

**Methods:**

Forty eligible patients with pathologically confirmed ESCC of clinical T3N + M0 stage were allocated to receive neoadjuvant immunotherapy (tislelizumab, q3w × 2 cycles) plus chemotherapy (nad‐paclitaxel + carboplatin, q3w × 2 cycles) and node‐sparing radiotherapy (41.4 Gy/23 times) treatment. The primary end point of this study is the pathological complete response rate. The secondary end points include major pathological response rate, adverse events, 2‐year overall survival, and disease‐free survival.

**Discussion:**

This is the first prospective clinical trial to investigate the safety and efficacy of NCIT combined with node‐sparing radiotherapy for clinical T3N+ locally advanced ESCC. We hypothesize that this promising strategy can provide a better pCR rate and acceptable safety.

**Trial Registration:**

ClinicalTrial.gov: NCT06965829

## Graphical abstract

The CINSREC trial is the first prospective clinical trial to investigate the safety and efficacy of NCIT combined with node‐sparing radiotherapy for clinical T3N+ locally advanced ESCC. This strategy may provide a better pCR rate and acceptable safety.

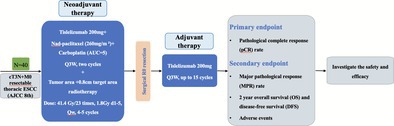

## 1 Introduction

In the world, esophageal cancer ranks as the sixth most frequent cause of death from cancer [1]. Each year, China accounts for nearly half of the new cases globally, and the main histological type is esophageal squamous cell carcinoma (ESCC) [2]. When most ESCC patients are first detected, they are already in a locally advanced stage, and surgery alone is far from sufficient due to high recurrence or metastasis rates [3].

In recent years, for the multidisciplinary treatment of locally advanced ESCC, the most promising research area is neoadjuvant chemoimmunotherapy (NCIT) [4, 5, 6, 7]. A multicenter real‐world study also verified the safety and effectiveness of NCIT in ESCC [8]. However, in clinical practice, it is not uncommon to encounter cases with severe tumor invasion, such as cT3 stage. The tumor regression is not satisfactory after NCIT sometimes, reflecting the insufficient efficacy of NCIT for advanced T‐type tumors. Therefore, it might be necessary to add concurrent radiotherapy to NCIT for improving the local control effect, while it also means higher toxicity [9]. Additionally, research has shown that patients with locally advanced ESCC undergoing chemoradiotherapy, when radiating more than 50% tumor‐draining lymph nodes (TDLNs), which is crucial for the production of tumor‐specific CD8^+^ effector T cells [10], can lead to immune suppression and poor overall survival [11]. The preoperative node‐sparing radiotherapy combined with NCIT, which means precisely irradiating the tumor area without TDLN, is expected to further enhance the therapeutic efficacy of NCIT and reduce the treatment‐related toxicity.

This study aims to explore the safety and efficacy of NCIT combined with node‐sparing radiotherapy for locally advanced clinical T3N+ stage ESCC.

## 2 Methods

### 2.1 Study Design

**FIGURE 1:**
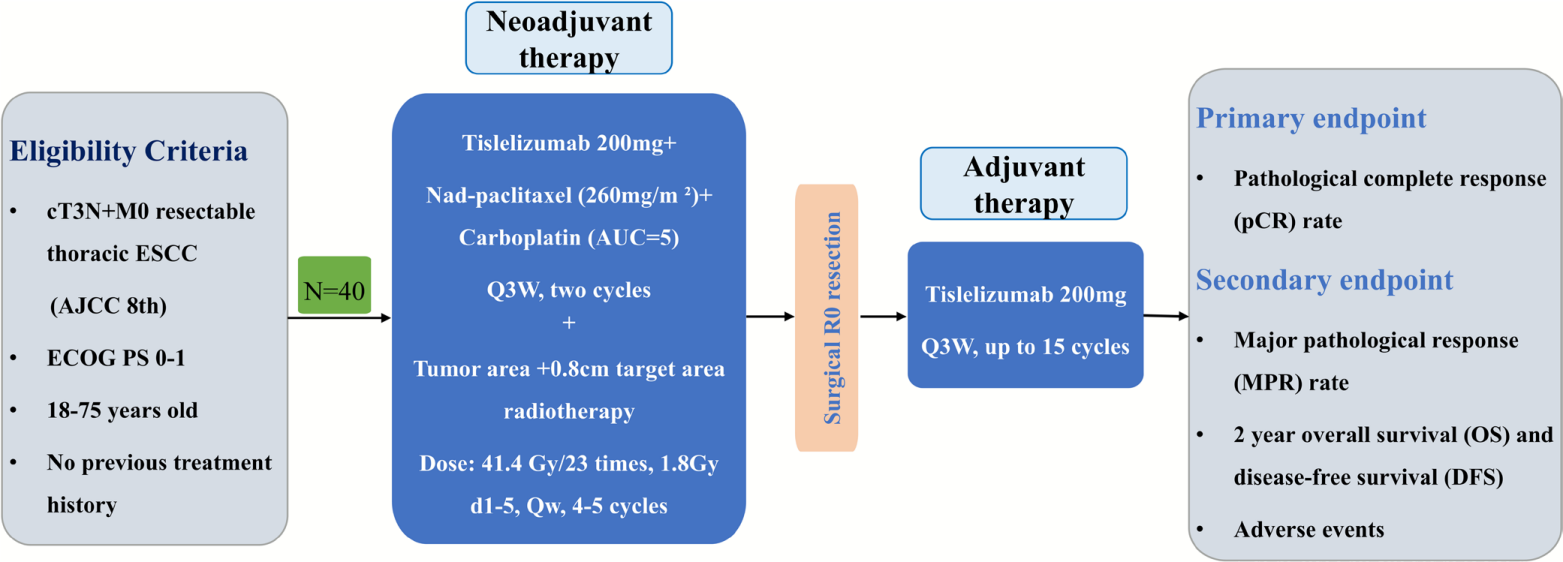
The flow chart of CINSREC study.

The CINSREC study was a prospective, single‐arm phase II clinical study aimed at observing the safety and efficacy of NCIT combined with node‐sparing radiotherapy for clinical T3N+ stage locally advanced ESCC. The primary end point was the pCR rate. The secondary end points include major pathological response (MPR) rate, adverse events, 2‐year OS, and DFS. pCR was regarded as the nonexistence of viable tumor cells in both the main tumor area and all dissected lymph nodes. Tumor response to preoperative therapy was assessed using the Chirieac Tumor Regression Grade (TRG) system, which classifies response into four categories [12]: TRG1 (no residual tumor), TRG2 (< 10% residual tumor), TRG3 (10%–50% residual tumor), and TRG4 (> 50% residual tumor). TRG1 and TRG2 are regarded as MPR. The investigator assessed recurrence as the emergence of one or more new lesions. Figure 1 shows the study flow chart. The study began on July 1, 2025, and the date set to be completed is July 1, 2028.

### 2.2 Study Procedures

Eligible patients agreed to informed consent before enrollment and were assigned to receive NCIT combined with node‐sparing radiotherapy followed by esophagectomy. All patients received neoadjuvant immunotherapy (tislelizumab 200 mg d1, q3w × 2 cycles) plus chemotherapy (nad‐paclitaxel 260 mg/m^2^ d1 + carboplatin AUC = 5 d1, q3w × 2 cycles) and concurrent node‐sparing radiotherapy (41.4 Gy/23 times: 1.8 Gy d1–d5, qw × 4–5 cycles). According to RECIST 1.1 [13], the efficacy was appraised. Surgery was performed on patients after 4–6 weeks of the last treatment. For patients whose disease progression was confirmed and surgery was not feasible, after multidisciplinary treatment discussions, definitive chemoradiotherapy and other drug treatments were adopted. Additionally, for patients who had experienced severe adverse events to treatment, a multidisciplinary discussion was also required to decide whether to continue with the subsequent treatment as planned or to abandon the surgery. All patients enrolled, including those who dropped out due to different reasons, will be included in the intention‐to‐use analysis. According to the patients' desires, those who had R0 resection always received adjuvant immunotherapy (tislelizumab 200 mg q3w) for up to 1 year after the surgery. Patients who did not have an R0 resection received adjuvant radiotherapy.

If any serious adverse events (SAEs) occur, treatment can be interrupted or postponed, and treatment can be resumed until the established recovery treatment standards are met. Safety‐related end points include neoadjuvant treatment‐related adverse events (TRAEs), immune‐related adverse events (irAEs), resection rate, and surgical complications. TRAEs and irAEs were evaluated according to the American Cancer Institute Common Terminology Criteria for Adverse Events 5.0 (CTCAE5.0) [14]. SAEs are defined as Levels 3–5 TRAEs. During the trial period, if any serious adverse situations arise, the researcher needs to take immediate and proper preventive actions or halt the treatment right away. They are required to report to the principal researcher within 24 h and also complete and sign the report form regarding such serious adverse situations. The proportion of patients who have surgical excision in relation to the planned treatment group is known as the resection rate. All investigators were required to follow the standard operating procedures for drug management in the study, and surgical complications within 30 days after surgery were coded with the Clavien–Dindo classification [15].

Thoracoscopy combined with laparoscopic‐assisted esophagectomy was carried out on the selected patients by using McKeown surgery, which involved thoraco‐abdominal two‐field lymph node dissection. The lymph nodes are classified into the following types [16]: nodes related to the left recurrent laryngeal nerve, right recurrent laryngeal ganglion, nodes around the lower aortic arch, esophageal lymph nodes in the upper, middle and lower thorax, the lower prominence node, posterior mediastinal lymph nodes, pericardial lymph nodes, nodes along the lesser curvature, left gastric lymph nodes, hepatic lymph nodes, splenic lymph nodes, and abdominal lymph nodes. It is necessary to excise the bilateral recurrent laryngeal nerve nodes. An incision was made beneath the xiphoid process to form a tubular stomach, and esophagogastrostomy was carried out via a left vertical neck incision along the front edge of the sternocleidomastoid muscle.

Furthermore, we have designed to collect tumor tissue and blood for translational research, which will significantly increase the study's scientific value.

## 3 Eligibility Criteria

### 3.1 Inclusion Criteria

1. The patient volunteers showed their willingness, signed a consent form, agreed to take part in the study, followed the follow‐up procedures well, and exhibited good compliance.
2. ESCC diagnosed by histopathology.
3. The patients have not received any systemic or local treatment for esophageal cancer in the past.
4. The patients were evaluated as thoracic esophageal cancer through auxiliary examinations and clinical staging as T3N + M0 (according to AJCC 8th edition of esophageal cancer [17]).
5. Expected to achieve R0 resection.
6. Both males and females aged between 18 and 75 are eligible.
7. The Eastern Cooperative Oncology Group (ECOG) performance status (PS) score is 0 or 1.
8. The patients planned to undergo surgical treatment after the neoadjuvant therapy was completed.
9. The patients had no surgical prohibitions.
10. The main organ functions of patients were normal, including: (a) blood routine examination (no blood‐related drugs were allowed to be used within 14 days before the first use of the study drug); Neutrophil count ≥ 1.5 × 10^9^/L, platelet count ≥ 100 × 10^9^/L, and hemoglobin ≥ 90 g/L; (b) Blood biochemistry test: Total bilirubin ≤ 1.5 × ULN, ALT ≤ 2.5 × ULN, AST ≤ 2.5 × ULN, Serum creatinine ≤ 1.5 × ULN, or creatinine clearance rate ≥ 50 mL/min; (c) Coagulation function: INR ≤ 1.5 × ULN, and APTT ≤ 1.5 × ULN; (d) Blood sugar: in normal range and/or with diabetes, the blood sugar was controlled in a stable state during treatment.
11. Females should have a negative result in serum or urine pregnancy test within 72 h prior to the initiation of study drug administration. They should take effective contraceptive methods (such as intrauterine devices, contraceptive pills, or condoms) during the trial period and for at least 3 months after the last drug administration; For male participants whose partners were women of childbearing age, effective contraceptive measures should be taken during the trial period and within 3 months after the last dose.

### 3.2 Exclusion Criteria

1. Some factors making resection impossible include tumors that are not removable, surgical contraindications preventing removal, or individuals who decline surgery.
2. Patients having supraclavicular lymph node metastasis.
3. Poor nutritional status, BMI < 18.5 kg/m^2^; If the patient's nutritional support is corrected before enrollment and evaluated by the principal investigator, enrollment can continue to be considered.
4. Those with a known history of being allergic to components of drug regimen.
5. Have received or are currently receiving any of the following treatments in the past. (a) Any radiotherapy, chemotherapy, or other anti‐tumor drugs targeting tumors; (b) Within 2 weeks prior to the first use of the investigational drug, immunosuppressive or systemic hormone therapy was being used to achieve immunosuppressive effects (dose > 10 mg/day prednisone or equivalent dose); In the absence of active autoimmune diseases, inhalation or topical use of steroids and corticosteroids at doses > 10 mg/day of prednisone or equivalent doses are allowed as substitutes for adrenal cortex hormones; (c) Received attenuated live vaccine within 4 weeks prior to the first use of investigational drug; and (d) Having undergone major surgery or suffered severe trauma within 4 weeks prior to the first use of investigational drug.
6. A history of having an immunocompromised state, such as being HIV‐positive upon testing, or having other immunocompromising diseases, or having a history of organ or allogeneic bone marrow transplantation.
7. Heart‐related clinical symptoms that are not well‐controlled, such as the following: (1) Heart failure of NYHA Class II or a higher class; (2) Unstable angina; (3) Myocardial infarction within 1 year; (4) Clinically significant supraventricular or ventricular arrhythmias that have not been clinically intervened on or are still not well‐controlled after clinical intervention.
8. Within 4 weeks prior to the first use of the investigational drug, there has been a severe infection (CTCAE > Grade 2), such as severe pneumonia requiring hospitalization, bacteremia, and infection complications; Baseline chest imaging examination suggests the presence of active pulmonary inflammation, symptoms and signs of infection within 14 days before the first use of study drug, or when there is a requirement for oral or intravenous antibiotic treatment, apart from the prophylactic use of antibiotics.
9. Within the first 4 weeks of randomization, participation in other drug clinical studies has occurred.
10. Subjects with current interstitial pneumonia or interstitial lung disease, or having other pulmonary fibrosis, bronchiolitis obliterans‐like organizing pneumonia, pneumoconiosis, drug‐related pneumonia, idiopathic pneumonia that might interfere with the assessment and management of immune‐related pulmonary toxicity, or those whose screening CT shows active pneumonia or severe lung function impairment, and those with active pulmonary tuberculosis.
11. Patients with any active autoimmune condition or a history of autoimmune disease which might recur (autoimmune hepatitis, lung‐related interstitial conditions, eye‐related uveitis, enteritis, pituitary inflammation, vasculitis, nephritis, hyperthyroidism, and hypothyroidism); Patients having skin diseases not needing systemic treatment, such as leukoplakia, psoriasis, alopecia, patients with type I diabetes controllable by insulin treatment, or patients with a history of asthma that has completely subsided in childhood and requires no intervention can be part of the group; and Asthma patients cannot be included in the study.
12. There is active hepatitis B (HBV DNA ≥ 10^4^ copies/mL) and hepatitis C (HCV RNA is higher than the detection limit).
13. Diagnosed with other malignant tumors within 5 years prior to the first use of investigational drug, unless there is a low‐risk metastasis or death risk malignant tumor (5‐year survival rate > 90%), such as adequately treated basal cell carcinoma, squamous cell carcinoma, or cervical carcinoma in situ, may be considered for inclusion.
14. Pregnant or lactating women.
15. According to the researchers' assessment, there exist additional factors that might result in the forced ending of the study. These can have other severe ailments (involving mental disorders) which demand concurrent treatment, alcohol misuse, drug abuse, family or social aspects, all of which could influence the subjects' safety or compliance.

### 3.3 Pathologic Examination

Two senior pathologists reviewed the pathological report, which covered aspects such as the infiltration depth of the primary lesion, the histological type, the pathological conditions of margins, and the peripheral affected lymph nodes. The assessment of TRG and ypTNM staging was carried out.

### 3.4 Ethics Approval and Consent to Participate

The Ethics Committees of Shanghai Chest Hospital approved this study protocol (No. IS25103) in line with the Declaration of Helsinki [18]. Before being enrolled, all patients are required to give their informed consent.

### 3.5 Follow‐Up

The first follow‐up took place 1 month after the surgery. Since then, follow‐ups took place every 3 months within the first 2 years. Standard laboratory tests, chest enhanced CT scans, as well as neck and abdominal ultrasound examinations, are all included in the detailed examination items.

During the treatment period, if the patient has recurrence signs (such as relevant clinical manifestations), extra tumor assessment will be carried out; possible reoperation and further cancer treatment need to be recorded. During the follow‐up period without tumor recurrence, the use of other cytotoxic drugs is not allowed. Follow up on the patient's recurrence and survival status until the patient's death or the last known date of survival.

### 3.6 Statistics

The main end point of this study was pCR. According to reports from a large real‐world study [8], the pCR of NCIT was 23%, which served as a historical control for this study. After adding radiotherapy, the pCR rate was expected to increase to 43%, with a unilateral error rate of 0.05. Based on the binomial exact test, the test efficacy for detecting statistically significant differences between the ORR of the experimental group and historical values was 80%, with a sample size of 34 cases. A total of 40 patients needed to be recruited, assuming the dropout rate was 10%.

The 2‐year DFS and OS of patients were calculated by using the Kaplan–Meier method. Independent survival factors were assessed with the Cox proportional hazards model. Mean and standard deviation (SD) were utilized to represent continuous variables. Percentages were used to express categorical variables, which were compared by means of chi‐square tests. Fisher's exact test was utilized to assess the difference in proportion between the two groups. Nonparametric datasets are compared with a Wilcoxon test. The SPSS (version 24.0, Chicago, IL, USA) statistical analysis software program was utilized for all statistical analyses. Statistical significance will be considered when the *p* value is less than 0.05.

## 4 Discussion

In recent years, immunotherapy has gradually changed the traditional pattern of neoadjuvant therapy for esophageal cancer. Multiple phase II clinical studies have confirmed that NCIT is safe and effective in treating locally advanced resectable esophageal cancer, achieving a pCR rate of 16.7%–55.6% [4, 5, 7]. ESCORT‐NEO, as the first phase III clinical trial of neoadjuvant immunotherapy, suggested that NCIT has good safety and a pCR rate of up to 28.0% [19]. In addition, several retrospective studies suggest that NCIT has a greater advantage in reducing postoperative distant metastasis [20, 21, 22], and can achieve comparable or even better long‐term survival compared to neoadjuvant chemoradiotherapy (NCRT). A recently published large sample multicenter real‐world study showed that NCIT achieved better 2‐year OS (81.3% vs. 71.3%) and DFS (73.9% vs. 63.4%), and also showed a lower overall recurrence and metastasis rate (23.7% vs. 35.7%) than NCRT [8]. A meta‐analysis also confirmed the safety and efficacy of NCIT in ESCC, compared to NCRT [23]. Entering the era of immunity, NCIT has become the most widely used neoadjuvant therapy for locally advanced resectable ESCC in China. The ongoing comparison of NCIT and NCRT prospective, randomized controlled trials includes NICE2 and Keystone002 [24, 25].

However, in clinical practice, a situation is often encountered where the tumor invades severely. Even after receiving NCIT, the regression is not good, reflecting the insufficient effectiveness of NCIT for advanced T‐type tumors. Concurrent radiotherapy might be needed to further improve the local control effect. Several studies have confirmed that the addition of preoperative radiotherapy could significantly improve the regression of cT3–4 stage tumors. In the pre‐immune era, JCOG1109 and CIMISG1701 studies showed that NCRT can significantly increase the percentage of pathological T0 for cT3–4 stage tumors compared to neoadjuvant chemotherapy [26, 27]. After entering the era of immunotherapy, both PALACE‐1 [28] and NEOCRTEC1901 [29] trials have confirmed that neoadjuvant chemoradioimmunotherapy (NCRIT) can achieve better tumor downstaging for cT3–4a ESCC. In addition, a multicenter phase II clinical study, which focuses on cT4b conversion surgery, showed that NCRT can achieve higher conversion rates compared to NCT [30].

At present, there are no mature phase III clinical studies comparing the efficacy differences between NCRIT and NCIT in the treatment of locally advanced esophageal cancer. In a meta‐analysis of NIT for esophageal cancer, He et al. proposed that NCRIT can bring better therapeutic effects, but compared to NCIT, it also means higher toxicity reactions [9]. How to balance the efficacy and side effects is a key issue in the era of immunotherapy. A more rigorous definition of the radiotherapy target area is one of the strategies to achieve precise treatment and reduce toxic side effects. Furthermore, radiotherapy sometimes not only fights against tumors and activates the immune microenvironment, but also has a destructive effect on the T cells and lymph node microenvironment around the tumor. TDLN are crucial for the production of tumor‐specific CD8^+^ effector T cells, which are one of the main sources of antigen‐specific CD8^+^ T‐cell production triggered by dendritic cells [10]. In mouse tumor models, researchers have found that combining node‐sparing radiotherapy and immunotherapy, PD‐L1 blockade targeting TDLN was enhanced by delivering progenitor‐type‐depleted T cells to the tumor site to enhance the anti‐tumor T‐cell immune response, thereby improving tumor control and mouse survival rate [31]. Therefore, in the era of immunotherapy, more precise design is needed for defining the radiotherapy target area of esophageal cancer. The preoperative node‐sparing radiotherapy is expected to further enhance the combined effect of radiotherapy and NCIT and reduce toxicity.

It is worth noting that the node‐sparing radiotherapy has achieved preliminary results in other solid tumors. Song and colleagues have conducted a prospective, phase II clinical study on locally advanced rectal cancer with NCIT combined with node‐sparing radiotherapy [32]. The preliminary results were reported at the ESMO IO conference in 2024, suggesting that radiotherapy only targeting the primary tumor can greatly improve the pathological remission of the primary tumor with pCR at 78.8%, and MPR at 90.9%, respectively.

In summary, we designed this CINSREC study to evaluate whether NCIT combined with node‐sparing radiotherapy can provide better tumor downstaging for clinical T3N+ ESCC. This study may help to answer the following questions: first, compared to NCIT, can the combination of NCIT and node‐sparing radiotherapy provide better tumor regression and higher 2‐year OS and DFS; second, whether this therapeutic strategy has an acceptable safety profile.

## Author Contributions

The study was conceived by X.F.G. as the chief investigator, who is also in charge of the overall conduct of the study. The study design and patient enrollment had the participation of X.W.C. The clinical input is the responsibility of Z.R.Y. and Y.Z. (Ya Zeng). The trial was initiated by X.Z., C.J.C., and X.F.G., and they also supervised the manuscript drafting. The translational analysis connecting clinical and basic research was contributed by L.Y.Z., Y.Z. (Yan Zhuo), and Y.K.W. The final manuscript has been read and approved by all the authors, who are willing to take responsibility for all aspects of the work.

## Conflicts of Interest

The authors declare no conflicts of interest.

## Data Availability

The datasets used during the CINSREC study are available from the corresponding author upon a reasonable request.
